# Enhancing highway transportation safety resilience during emergencies: A network-based analysis and assessment

**DOI:** 10.1371/journal.pone.0307233

**Published:** 2024-07-19

**Authors:** Xue Zhang, Yi Lu, Jie Wang, Yongzheng Qi

**Affiliations:** 1 School of Traffic &Transportation Engineering, Changsha University of Science and Technology, Changsha, Hunan, China; 2 College of Civil Engineering and Architecture, Jiangsu University of Science and Technology, Zhenjiang, Jiangsu, China; Southwest Jiaotong University, CHINA

## Abstract

In order to evaluate the impact of emergencies on the resilience of highway transportation, a resilience network hierarchical model of the highway transportation system was constructed by analyzing the formation and emergence process of safety resilience in the highway transportation system. Four layers of networks were divided, including highway network, transport network, traffic network, and emergency network. Combined with the network hierarchical model, a resilience evaluation index system was designed, and an assessment method for highway transportation systems based on the fuzzy analytic hierarchy process(FAHP) was proposed. Finally, a case study of a public health emergency in a region of Hunan was carried out. The results show that the proposed method for evaluating the safety resilience of highway transportation systems can better reflect the overall resilience under public health emergencies, which is consistent with the quantitative analysis results through the system resilience curve. It helps to accurately evaluate the safety resilience of the system. At the same time, this method has the advantages of flexibility and simplicity in solving unstructured decision-making problems of the system, which helps to improve the safety production management and safety resilience level of highway transportation systems. In the future, the scope of research scenarios and regions can be expanded, and further analysis of the evolution of safety resilience and the ability of resilience development in different stages under external disturbances can be conducted in order to further explore and optimize the resilience of the system.

## 1. Introduction

Frequent occurrences of natural disasters, accidents, public health emergencies, and social security incidents have seriously disrupted the safe operation of urban systems and have significantly impacted people’s production, daily life, and social and economic development. As an essential channel for secure transportation amongst modern cities, the highway transport system plays a crucial role in national security and economic development. The impact of sudden events not only hinders highway transportation’s ability to fulfill its role in supporting and guaranteeing urban functions but has also had a profound effect on its own development. Therefore, it is essential to evaluate the safety resilience of the highway transportation system in the face of sudden events, analyze the evolving characteristics of its resilience, and lay a foundation for constructing resilient highway transport systems and cities.

The majority of research on the impact of sudden events on highway transport has focused on flooding [1, 2], earthquakes, accidents [3], and disasters, while public health emergencies have been given less attention [4–6]. Compared with other sudden events, the impact of public health emergencies on highway transportation and the societal economy is much more far-reaching. For example, the outbreak of COVID-19 at the beginning of 2020 spread rapidly across the country and even globally, resulting in extensive impacts on society, such as suspension of work and school, lasting over a prolonged period, and causing highway transport systems to shut down or be seriously impacted. The safety resilience of the highway transportation system was therefore greatly affected, and its performance was significantly reduced [7–11]. It is of great significance to evaluate the safety resilience of the highway transportation system during such sudden events, to determine the strength level, and to analyze the evolving special and temporal characteristics to better understand the current social environment.

In recent years, research on transportation system resilience has continuously grown and made significant progress in emergency response and disaster management. The research on resilience has gone through three stages: conceptual framework [12], index system [13, 14], and quantitative evaluation [15]. Huang et al. [16, 17] have constructed a conceptual model of system safety resilience from three dimensions: safety resilience function, elements or subsystems, and correlation representation. Murray-Tuite [18] has regarded resilience as a network characteristic of how a transport network can operate normally in cases of extreme disasters and has divided the transport network resilience into ten indicators: cooperation, redundancy, diversity, efficiency, safety, self-organization, strength, adaptability, fluidity, and the ability to recover quickly. Labaka et al. [19] have constructed a framework for the evaluation of critical infrastructure safety resilience from four dimensions: technology, organization, economy, and society, further indicating that the improvement of critical infrastructure resilience is of vital importance to public safety management. Joerin et al. [20] have suggested that the assessment of safety resilience should fully consider the socio-physical environment of the evaluation object, proposing to incorporate the socio-physical environment as one of the dimensions for safety resilience evaluation. Ahern et al. [21] have proposed considering four dimensions, i.e., multifunctionality, redundancy, modularity, and diversity, when evaluating urban safety resilience. Battelle [4] has utilized the resilience characteristic of the available system to measure the resilience of transportation systems, which can still ensure systems to compensate for losses and maintain their original functions when disturbed. Bruneau et al. [12] have put forward a classical resilience concept framework that resilience is defined as “the ability of a system to reduce the impact of disasters and maintain its own functions”, which has laid the foundation for quantitatively evaluating resilience by representing it as a “resilience triangle” enclosed within a system infrastructure functional curve and a time axis (the horizontal coordinate). Numerous scholars have proposed quantitative methods for evaluating system resilience based on such a framework. Omer et al. [22] have proposed a quantitative method for measuring the resilience of telecommunication network systems based on the research findings of Bruneau et al., defining the ratio of the network’s transmission value before and after disasters as the resilience of telecommunication networks. Ouyang et al. [23] have developed an indicator-based and evaluation framework for quantifying the resilience of infrastructure, which has multi-risk applicability and incorporates dynamic evolution factors, making it convenient for calculating the potential resilience of a system in the future. In terms of resilience evaluation methods, the Fuzzy Analytic Hierarchy Process is a relatively mature evaluation method that is widely used in many research fields, such as in the evaluation of innovation incubation ability of makerspaces [24], quantitative evaluation of social media privacy security [25], evaluation for effectiveness of national defense science and technology strategy [26], risk assessment on sponge city construction [27], safety risk assessment of gas environment in power foundation pits [28], safety resilience assessment of urban road traffic systemunder rainstorm waterlogging [2] and so on.

These studies have enriched the theory and practice of system safety resilience evaluation, yet they still require in-depth research on the construction of safety resilience models for highway transportation systems during emergencies, especially public health emergencies.

In view of this, this paper intends to construct a network hierarchical model of safety resilience for highway transport systems based on the analysis of the forming dimensions and the emergence process of safety resilience, design a safety resilience evaluation index system for highway transportation systems, and further propose evaluation method based on fuzzy analytic hierarchy process; By constructing a system resilience curve and analyzing examples, the effectiveness of the resilience evaluation method is verified, with the aim of accurately evaluating the safety resilience of highway transportation systems and improving the management ability and resilience level of system safety.

Accordingly, the contributions of this paper are mainly reflected in the following two points: (1)Analyze the forming dimensions and emergence process of safety resilience in highway transportation systems, combined with the factors related to safety resilience in highway transportation systems, construct a network hierarchical model of safety resilience for highway transportation systems, including highway network, transport network, traffic network, and emergency network; (2) Based on the network hierarchical model, a safety resilience evaluation index system for highway transportation systems is designed, and an evaluation method of safety resilience based on fuzzy analytic hierarchy process is proposed, whose effectiveness is verified.

## 2. Formation of safety resilience in the highway transportation system

**Fig 1:**
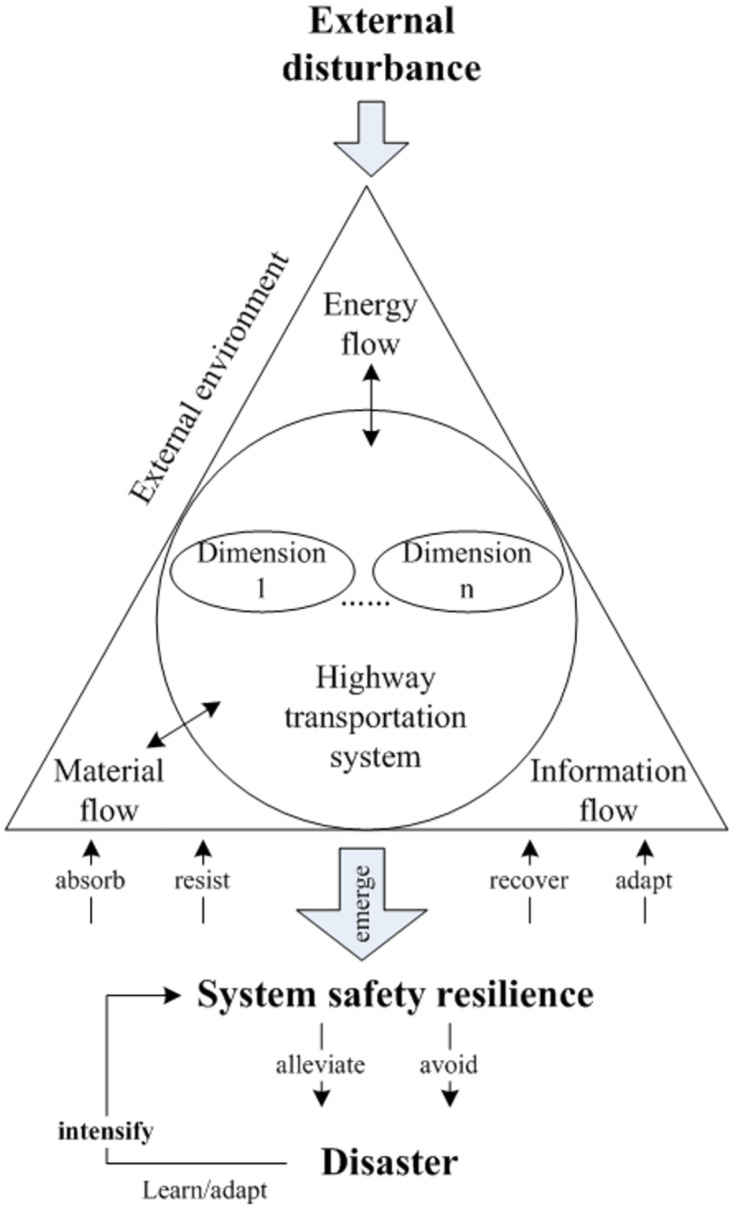
The resilience-shaped process of the highway transport system.

Resilience in the highway transportation system is a characteristic that emerges when the system encounters external disturbances. The definition and essence of resilience in the highway transportation system can be understood from the perspectives of system dimensions, safety resilience attributes, and resilience manifestation processes. The theoretical model illustrated in Fig 1 depicts the process of shaping the resilience of the highway transportation system.

### 2.1 Dimensions of resilience formation

**Fig 2:**
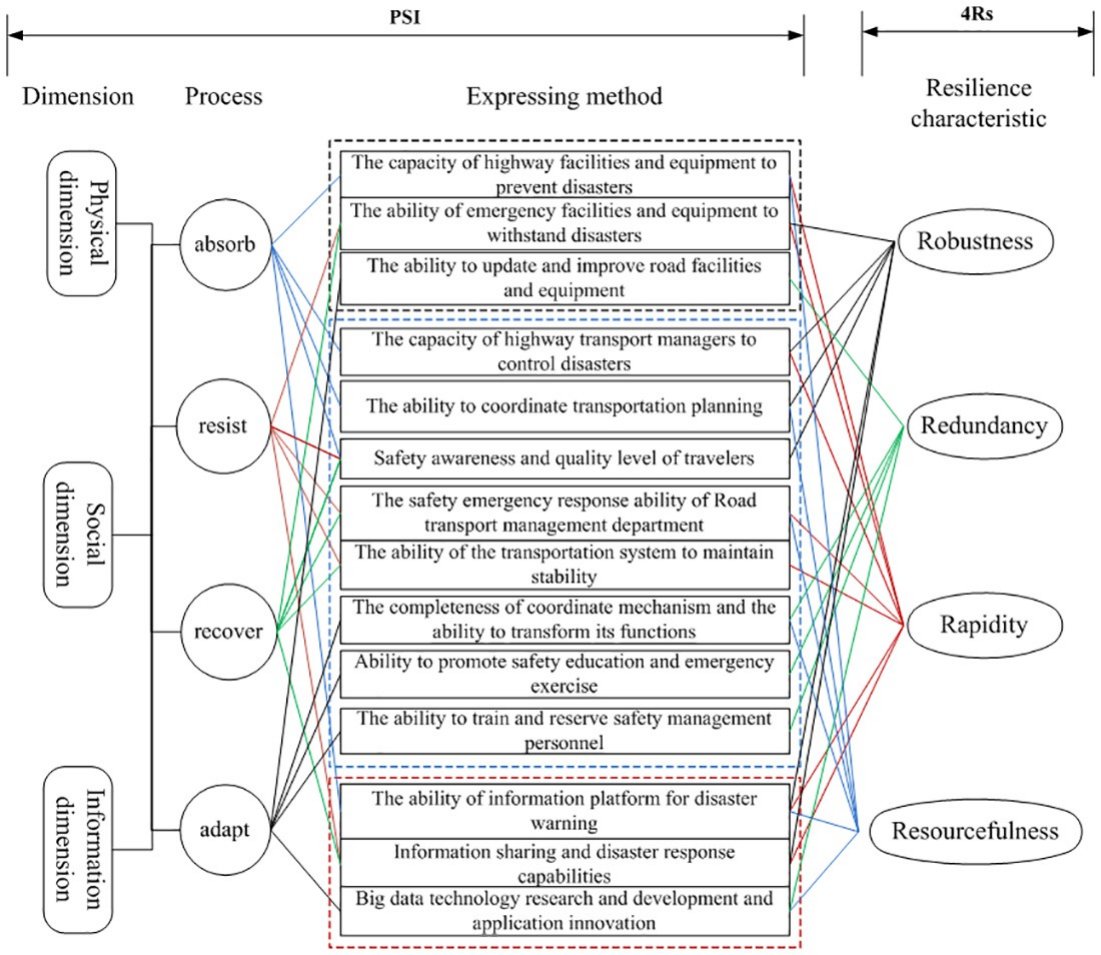
The analysis framework for forming dimension of safety resilience of highway transport system.

Considering the concept of the “three-dimensional space” [29] in urban settings, the highway transportation system can be seen as a combination of physical, social, and information dimensions. The physical dimension constitutes the foundation of the transportation system and includes infrastructure such as roads, bridges, and vehicles. The social dimension represents the driving force behind the development of the transportation system and encompasses drivers, passengers, pedestrians, road construction, and management activities. The information dimension connects the physical and social dimensions, involving the exchange of information between individuals, between individuals and devices, and between devices. It also encompasses information technology and digital technology. The system’s resilience in the “three-dimensional space” can be categorized into three dimensions: physical, social, and information (referred to as PSI). Based on existing research, the attributes of safety resilience in the system mainly include four aspects: 1) Robustness, which refers to the system’s ability to function normally in the face of external risks and disturbances; 2) Redundancy, which refers to the ability of the system’s key components to be replaced in the event of external risks, indicating the degree of resource surplus; 3) Rapidity, which refers to the speed at which the system recovers to its pre-event state after being affected by external influences; 4) Resourcefulness, which refers to the system’s ability to identify problems, establish priority orders, and mobilize resources in the event of a disaster. These attributes correspond to the 4Rs resilience attributes proposed by Bruneau. By combining the PSI dimensions in the three-dimensional space and the 4Rs resilience attributes, a dimensional analysis framework for forming safety resilience in the highway transportation system can be constructed, as shown in Fig 2.

### 2.2 Emergence process of resilience

**Fig 3:**
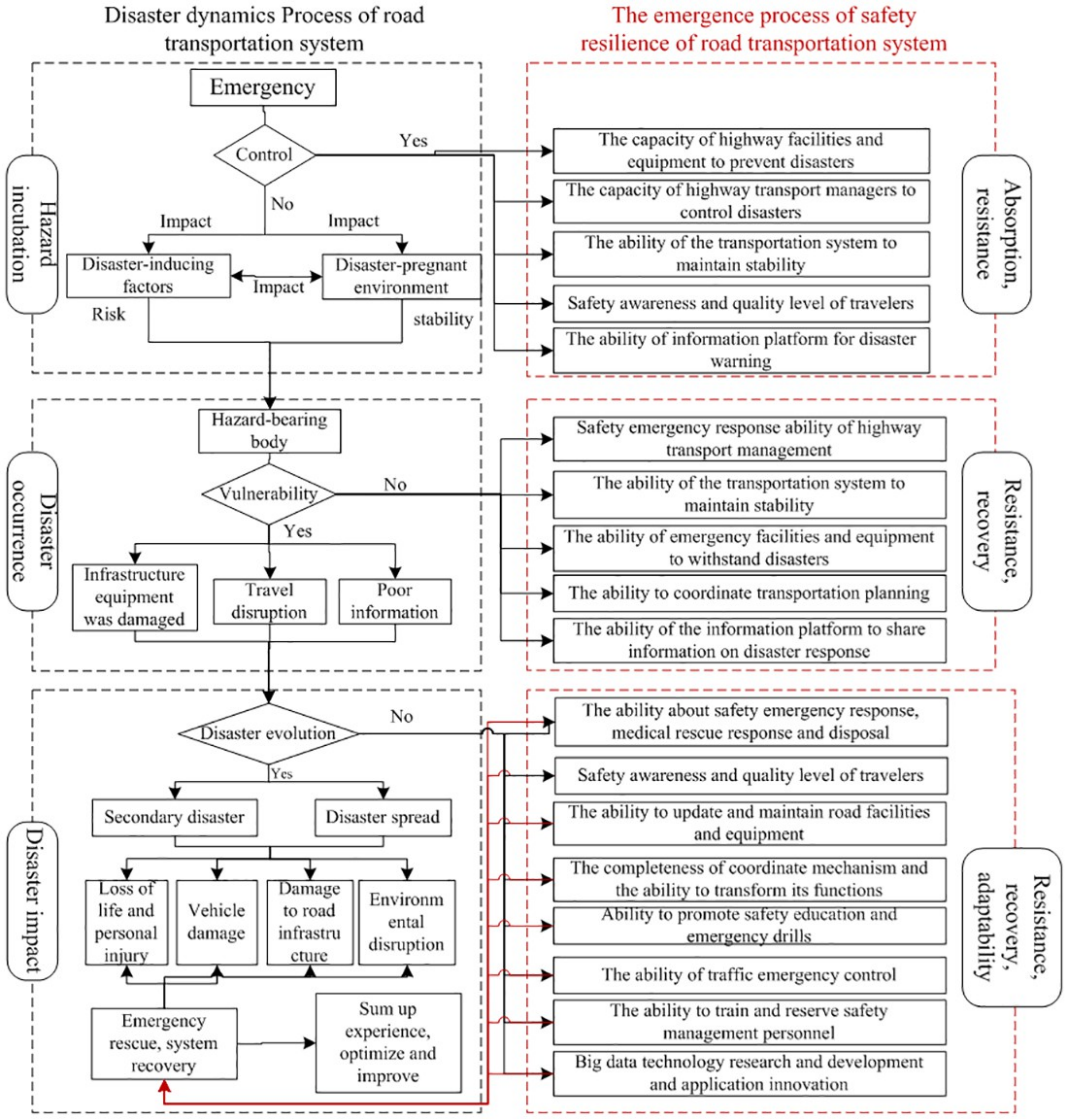
Mergence mechanism of safety resilience in highway transport system.

The highway transportation disaster system is part of a regional disaster system. Its existence relies on and is influenced by all factors and their combinations in the region that contribute to or inhibit highway transportation disasters. As part of the regional disaster system, the highway transportation disaster system consists of three subsystems: hazard factors, vulnerable elements, and the hazard-prone environment. According to disaster dynamics, a complete highway transportation disaster undergoes three phases: hazard incubation, disaster occurrence, and disaster impact. The emergence of safety resilience is closely related to the disaster process. Therefore, the emergence process of safety resilience in the highway transportation system can be analyzed based on these three phases: hazard incubation, disaster occurrence, and disaster impact (see Fig 3).

During the hazard incubation phase, the highway transportation system faces risks and disturbances due to sudden events, resulting in an unstable hazard-prone environment and the mutation of hazard factors, making the system vulnerable. The combined effects of hazard factors and the hazard-prone environment lead to the occurrence of disasters. If the risks and disturbances resulting from sudden events are not effectively controlled, disasters will occur. These disasters further affect the vulnerable elements, exacerbating the destructive force of disasters. This leads to damage to the highway transportation infrastructure, disruptions in people’s travel, and impaired transportation and communication. If the system’s emergency response capacity is inadequate, the disaster will continue to escalate. As time passes and the scope of the disaster expands, a series of secondary disasters will occur, affecting the highway transportation system and resulting in severe consequences such as damage to road facilities and vehicles, environmental destruction, and casualties. Throughout this process, the system continues to resist the destructive impact of the disaster, strives to restore itself to a normal state, learns from the experience of responding to emergencies, optimizes its safety status, enhances its safety resilience, and prepares better for the next risk disturbance.

## 3. Materials and methodology

### 3.1 Materials

At the beginning of 2020, the COVID-19 pandemic broke out and swept the world, which had a huge impact on the highway transportation system and affected the industry to varying degrees after the outbreak. As a critical link in epidemic prevention and control and an important lifeline for personnel and material transportation, the highway transportation system was most affected by the epidemic. Hunan Province, a province in central China that connects the north and south with a high traffic volume, was significantly affected by the epidemic. During the initial outbreak period (January-February 2020), compared with the same period in 2019, the passenger turnover of highway transportation in Hunan Province decreased by 50.02%, the passenger turnover decreased by 53.35%, the freight volume decreased by 14.5%, and the freight turnover decreased by 20.98%. This article selects highway transportation during the first wave of the epidemic in early 2020 and the rebound of the epidemic on July 28, 2021, as the analysis case.

### 3.2 Methodology

In this section, a safety resilience model of highway transportation systems to qualitatively evaluate resilience values was constructed. A quantitative measurement of resilience was introduced to validate the model’s effectiveness.

#### (1) Construction of the safety resilience model for the highway transportation system

**Fig 4:**
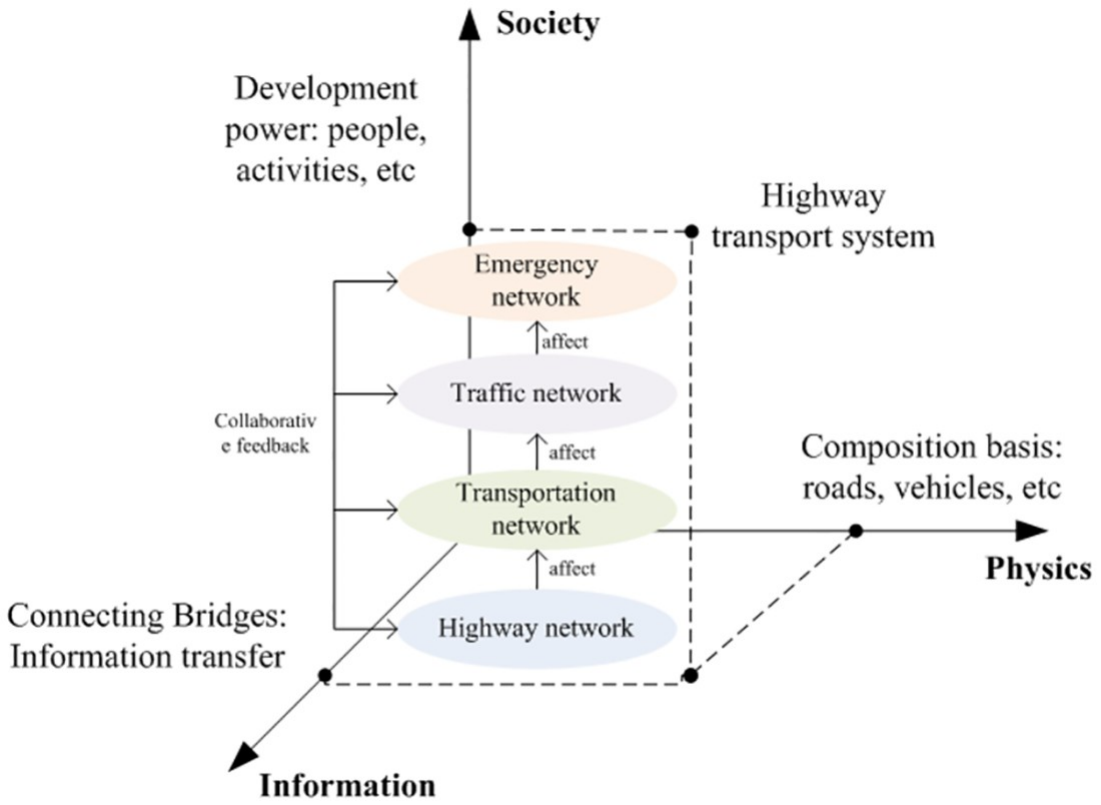
A network hierarchically model of safety resilience for the highway transportation system.

Based on the process of forming safety resilience in the highway transportation system, combined with the factors influencing safety resilience and the division of the system into the three-dimensional space, the highway transportation system can be regarded as the integration of a four-layered network: highway network, transportation network, traffic network, and emergency network. The highway transportation system’s safety resilience network model can be constructed hierarchically, as depicted in Fig 4.

In Fig 4, the highway network is positioned at the lowest level within the system and serves as the foundation for smooth operation. It ensures the proper functioning of highway transportation by considering factors such as the performance of road infrastructure, traffic capacity, maintenance capabilities, and emergency resource allocation and positioning.

The traffic network sits above the highway network and is directly influenced by it. In the event of damage to the highway network, the traffic network would be unable to function correctly, thereby impacting the traffic capacity and overall stability of the transportation system.

The transportation network is located above the traffic network and is constrained by the highway network and traffic network. Both directly affect the transportation capacity of the transportation network. Moreover, the efficient operation of the transportation network directly impacts the system’s safety resilience, including transportation management, security measures, and disaster prevention capabilities.

Finally, the emergency network occupies the highest level within the entire network. Its primary function is to coordinate emergency relief efforts during emergencies. It also promotes interdepartmental and interagency collaboration during regular times, emphasizing disaster prevention, mitigation, and early warning systems.

Combining the formation and emergence process of safety resilience in highway transportation systems and the hierarchical integration model of safety resilience networks in highway transportation systems, and referring to the evaluation index system for urban disaster resilience evaluation in reference [30], 16 indicator items for the evaluation of safety resilience of highway transportation systems during emergencies were determined (see Table 1). Based on the fuzzy analytic hierarchy process, the safety resilience of the highway transportation system in Hunan Province during the epidemic were evaluated. The specific process is as follows:

**Table 1:** Safety resilience index system of highway transport system.

Assessment Objectives	Primary Indicators	Secondary Indicators
Safety Resilience of Highway Transportation System	Highway Network T_1_	Highway Traffic Capacity; Highway Maintenance Capability; Layout of Emergency Resources and Rescue Sites; Performance of Highway Facilities
	Transportation Network T_2_	Highway Transportation Security Capability; Highway transportation Management Capability; Highway transportation Capability; Highway Disaster Prevention Capability
	Traffic Network T_3_	Traffic Planning and Coordination Capability; Construction of Traffic Information Platform; Stability of Traffic System; Traffic Emergency Control Capability
	Emergency Network T_4_	Coordination Mechanism Completeness; Funding and Material Support Capability; Government Function Transformation Capability; Mobilization Capability of Social Forces.

Using a scale of 1–9, ten experienced experts in the field of highway transportation safety management evaluated the established resilience indicators through pairwise comparison and matrix calculations, as shown in Table 2.

**Table 2:** Judgment matrix of the primary index of highway transport system.

***T***	***T***_**1**_	***T***_***2***_	***T***_**3**_	***T***_**4**_
***T***_**1**_	1	1/3	1/2	1/5
***T***_***2***_	3	1	4	2
***T***_**3**_	2	1/4	1	1/4
***T***_**4**_	5	1/2	4	1

Matrix T used for judgment:

$$T=\left[\begin{array}{cccc} 1 & 1/3 & 1/2 & 1/5\\ 3 & 1 & 4 & 2\\ 2 & 1/4 & 1 & 1/4\\ 5 & 1/2 & 4 & 1 \end{array}\right]$$

Similarly, the judgment matrix for highway network:

$$T_{1}=\left[\begin{array}{cccc} 1 & 7 & 4 & 3\\ 1/7 & 1 & 1 & 1/2\\ 1/4 & 1 & 1 & 1\\ 1/3 & 2 & 1 & 1 \end{array}\right]$$

Judgment matrix for the transportation network:

$$T_{2}=\left[\begin{array}{cccc} 1 & 3 & 1/2 & 1\\ 1/3 & 1 & 1/2 & 1/3\\ 2 & 2 & 1 & 1/2\\ 1 & 3 & 2 & 1 \end{array}\right]$$

Judgment matrix for the traffic network:

$$T_{3}=\left[\begin{array}{cccc} 1 & 1/3 & 1/3 & 1/3\\ 3 & 1 & 1/2 & 1\\ 3 & 2 & 1 & 1\\ 3 & 1 & 1 & 1 \end{array}\right]$$

Judgment matrix for the emergency network:

$$T_{4}=\left[\begin{array}{cccc} 1 & 2 & 2 & 3\\ 1/2 & 1 & 3 & 3\\ 1/2 & 1/3 & 1 & 2\\ 1/3 & 1/3 & 1/2 & 1 \end{array}\right]$$

The consistency ratio *C*_*R*_ and *λ*_max_ were calculated for the judgment matrix, as shown in Table 3.

**Table 3:** The result of the consistency test.

Judgment matrix	*C*_*R*_	*λ*_max_
***T***	0.0744	4.1988
***T***_**1**_	0.0212	4.0567
***T***_**2**_	0.0776	4.2072
***T***_**3**_	0.0227	4.0606
***T***_**4**_	0.0536	4.1431

The C_R_ value for each judgment matrix is less than 0.1, indicating that the consistency test has passed and the weight calculation process has consistency. The weight vector A for each secondary indicator for the evaluation target is (0.0152, 0.0507, 0.0088, 0.0120, 0.1257, 0.0479, 0.1139, 0.1587, 0.0114, 0.0292, 0.0415, 0.0343, 0.1435, 0.1129, 0.0580, 0.0364).

#### (2) Quantitative measurement method for resilience

**Fig 5:**
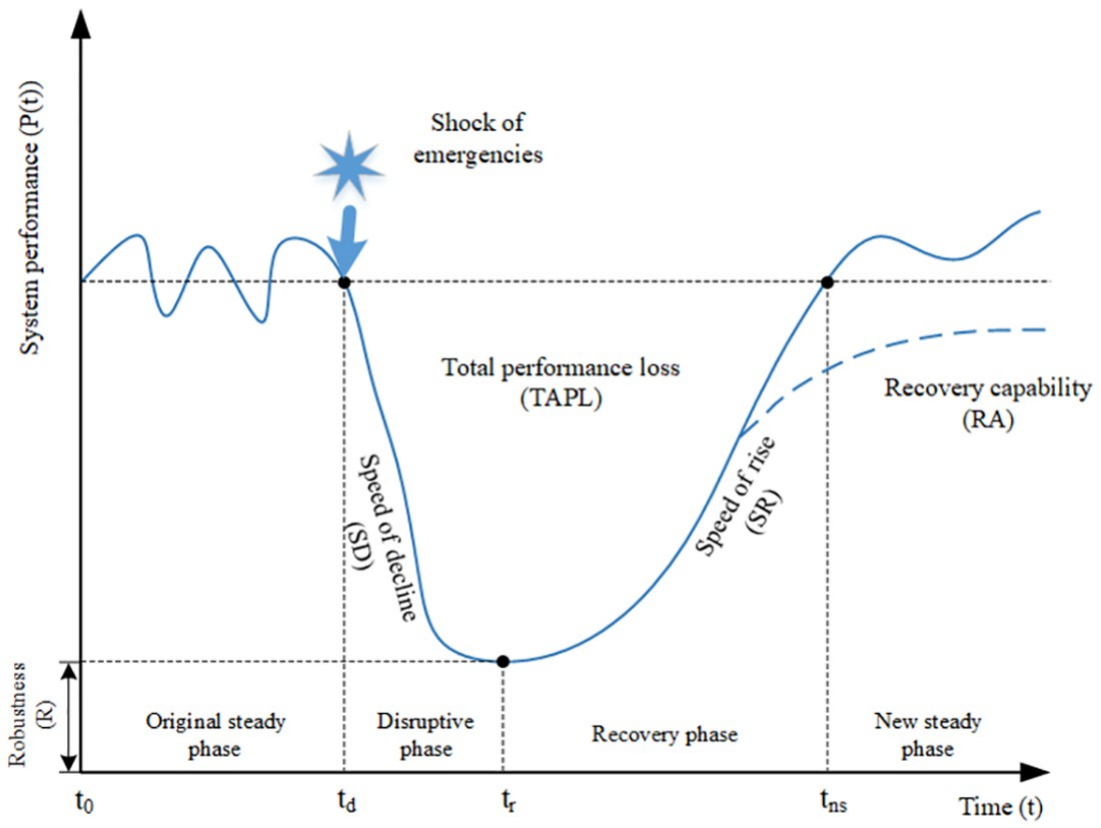
Resilience loss measurement based on the resilience triangle.

Based on the concept of the “resilience triangle” [12] and a quantitative evaluation framework for resilience [23], this study introduced a resilience quantitative assessment method [31, 32] proposed by Nan and Sansavini in 2017 for assessing the safety resilience of highway transportation systems before, during, and after disruptions caused by unexpected events. The proposed approach utilizes the “resilience triangle” to illustrate the impact of these events on the safety resilience of the highway transportation system, as shown in Fig 5. The y-axis represents system performance(P) in the figure, while the x-axis represents time. The curve, also referred to as the resilience curve, depicts the gradual decline and subsequent recovery of system performance after the disruptive event. Assuming that the disruptive event occurs at time t_d_, the system experiences a decrease in performance until time t_r_ when the recovery process initiates, continuing until time t_ns_ when the system reaches a new stable state. Considering these time points, the entire system development process can be divided into four phases.

The first stage is the initial stable phase (t < t_d_), representing the period before the system is disturbed. The performance level during this stage is often considered as the target value. Hence, the average performance value can be used as the benchmark for analysis.

The second stage is the disruptive phase (t_d_ < t < t_r_), where the system absorbs disturbances, and the performance begins to decline until reaching its lowest level. The robustness (R) can be used to describe the system’s capacity to absorb these disturbances: R = min{P(t)} (t_d_ < t < t_r_). From this, the maximum impact of the disturbance event on the system can be calculated as MI = Baseline-R. In addition, the system’s ability to absorb disturbances can be reflected through the speed of performance decline (SD) and the system’s performance loss during this stage (PL_DP_), which can be approximated as follows:

$$SD=\frac{P(t_{d})-P(t_{r})}{t_{r}-t_{d}}$$ (1)

The system’s performance loss during this stage can be calculated using the following formula:

$$PL_{DP}=\int_{t_{d}}^{t_{r}}\left(P(t_{0})-P(t\right))dt$$ (2)

The third stage is the recovery phase (t_r_ < t < t_ns_), where the system’s performance starts to rise until reaching a new stable state. The adaptive and recovery capability of the system during this process can be represented by the speed of performance improvement (SR) and the system’s performance loss during the recovery phase (PL_RP_), which can be approximated as follows:

$$SR=\frac{P(t_{\text{ns}})-P(t_{r})}{t_{ns}-t_{r}}$$ (3)

The system’s performance loss during this stage can be calculated using the following formula:

$$PL_{RP}=\int_{t_{\text{r}}}^{t_{ns}}\left(P(t_{0})-P(t\right))dt$$ (4)

The fourth stage is the new steady phase (t > t_ns_), in which the system’s performance reaches a new stable state and maintains at this level. It is worth noting that the new stable level may be consistent with the initial stable level, or may be higher or lower than it,. To reflect this change in the system, the system recovery ability (RA) can be introduced, which can be simply calculated as:

$$RA=\left|\frac{P(t_{ns})-P(t_{r})}{P(t_{0})-P(t_{r})}\right|$$ (5)

In the overall process, the total performance loss of the system can be represented by the variable “TPL”.


$$TPL=\text{P}L_{DP}+PL_{RP}=\int_{t_{d}}^{t_{ns}}(P(t_{\text{d}})-P(t))dt$$ (6)

The resilience of the system can be calculated using the following formula:

$$GR=\frac{P(t_{\text{d}})\times TI-\int_{t_{d}}^{t_{ns}}(P(t_{d})-P(t))dt}{P(t_{d})\times TI}$$ (7)

Where P(t_d_) represents the system performance before disturbance, and TI represents the total time that the system is affected by disturbances.

## 4. Result

### 4.1 Qualitative evaluation

The safety resilience evaluation of highway transportation systems was set as a five-level resilience evaluation from “strong” to “weak”, i.e., V = {strong, relatively strong, general, relatively weak, weak}. Twenty-nine personnel involved in highway transportation safety management (government department leaders, employees, researchers in scientific research institutions, university teachers, doctoral students, master students, etc.) were invited to evaluate each indicator and determine the resilience level based on objective data and quantifiable standards. A membership relationship matrix was constructed for the relationship between each indicator and its resilience rating, as shown in the matrix L:

$$L=\left[\begin{array}{ccccc} 0.1034 & 0.5517 & 0.3103 & 0.0345 & 0.0000\\ 0.0690 & 0.3103 & 0.3793 & 0.2069 & 0.0345\\ 0.0000 & 0.2069 & 0.4828 & 0.2069 & 0.1034\\ 0.1034 & 0.4483 & 0.3793 & 0.0690 & 0.0000\\ 0.2759 & 0.3793 & 0.3448 & 0.0000 & 0.0000\\ 0.3448 & 0.6552 & 0.0000 & 0.0000 & 0.0000\\ 0.1379 & 0.6207 & 0.2069 & 0.0345 & 0.0000\\ 0.3103 & 0.4828 & 0.2069 & 0.0000 & 0.0000\\ 0.1034 & 0.1379 & 0.3793 & 0.3103 & 0.0690\\ 0.3448 & 0.4828 & 0.1379 & 0.0345 & 0.0000\\ 0.1379 & 0.3793 & 0.3793 & 0.1034 & 0.0000\\ 0.1034 & 0.4138 & 0.3793 & 0.1034 & 0.0000\\ 0.1379 & 0.2069 & 0.3448 & 0.2414 & 0.0690\\ 0.0000 & 0.2759 & 0.5172 & 0.1724 & 0.0345\\ 0.0690 & 0.2414 & 0.3448 & 0.2069 & 0.1379\\ 0.2759 & 0.3103 & 0.3448 & 0.0690 & 0.0000 \end{array}\right]$$

Among them, the membership degree *l*_*vd*_(*t*_*ij*_) = *a*_*ijd*_/*n*, Where, t_ij_ represents each pair of secondary evaluation indicators(i,j = 1,2,…,4); a_ijd_ represents the frequency of indicator t_ij_ in the evaluation rating V_d_(d = 1,2,…,5); and n represents the number of evaluators.

Using the weight vector A to conduct fuzzy operations on matrix L, the fuzzy operator B was obtained, which shows the degree of membership of highway transportation system safety resilience in each evaluation level:

B=A•L=(0.1763,0.4010,0.3063,0.0924,0.0241)

From the perspective of assigning values to evaluation comments, the evaluation comment set V = (v_1_, v_2_, v_3_, v_4_, v_5_) = {strong, relatively strong, general, relatively weak, weak} = (1, 0.8, 0.6, 0.4, 0.2). Therefore, the overall fuzzy comprehensive evaluation of the safety resilience of the highway transportation system is:

R=B•V=0.7226

### 4.2 Quantitative measurement

**Fig 6:**
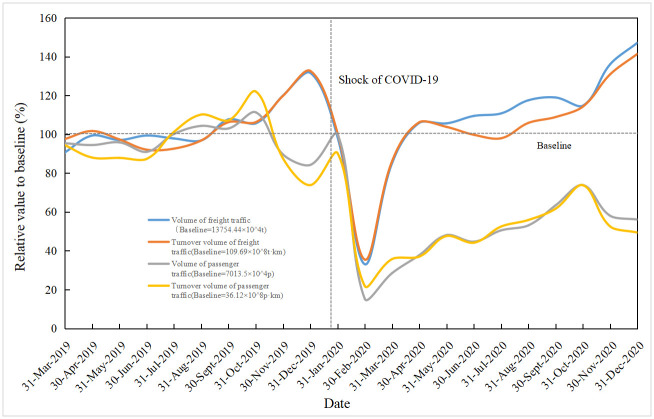
Highway transport resilience curve during the first wave of epidemic.

**Fig 7:**
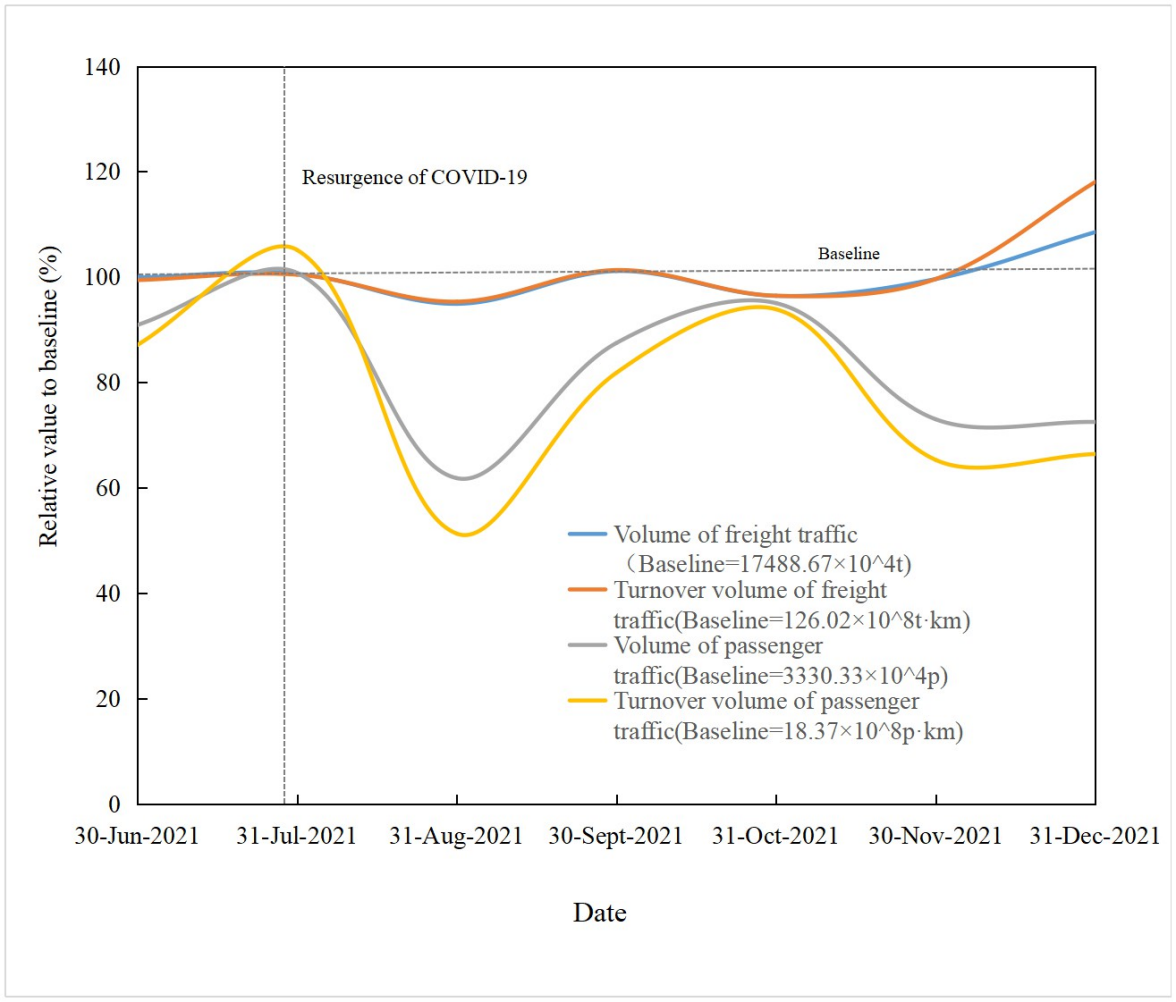
Resilience curve of highway transport during the rebound epidemic.

This study selected the following passenger and freight transport indicators to reflect the capacity of highway transportation: freight volume, freight turnover, passenger volume, and passenger turnover. Monthly data from the National Bureau of Statistics, Hunan Provincial Statistics Bureau, and the Ministry of Transport website were used. To compare the resilience among indicators with different dimensions, each indicator was normalized by comparing each indicator with its base value (the mean value of each index before the impact of the epidemic). Then the resilience curve of each index was drawn (see Figs 6 and 7).

Based on formulas (1)–(6) and the original statistical data, the resilience capacity of the system during the two waves of the epidemic can be obtained, as shown in Table 4.

**Table 4:** Detailed resilience metrics of the highway transportation system in Hunan Province during two epidemics.

	Index	R(%)	SD	SR	TPL	RA	TI
First wave of COVID-19 (January 23, 2020)	Freight volume	33.1	53.17	38.99	80.58	1	2.97
	Freight turnover	35.48	51.28	38.16	75.85	1	2.95
	Passenger volume	15.28	64.15	9.38	245.47	0.39	4.62
	Passenger turnover	21.87	51.02	7.27	194.32	0.38	4.62
Rebound of COVID-19 (July 28, 2021)	Freight volume	94.91	4.64	6.22	5.12	1	1.92
	Freight turnover	95.31	4.27	6	4.63	1	1.88
	Passenger volume	61.83	34.81	16.6	56.88	0.87	3.1
	Passenger turnover	51.3	44.4	21.28	74.66	0.87	3.1

### 4.3 Comparative analysis

Based on formula (7) and the data in Table 4, the system resilience of highway transportation in Hunan Province during the two waves of the epidemic can be calculated (see Table 5). By combining Figs 6 and 7 and Table 4, it can be observed that after the first epidemic, the system’s performance significantly declined, with decreases of 65.44%, 65.14%, 84.79%, and 68.43% in freight volume, freight turnover, passenger volume, and passenger turnover, respectively. Although the subsequent rebound epidemic also led to a decline in system performance, the magnitude was significantly reduced to 5.09%, 4.69%, 38.17%, and 48.7% for the respective indicators. This indicates an improvement in system resilience, which can also be observed from the changes in resilience before and after the various indicators. Considering the resilience of each indicator, the overall system resilience after the first epidemic and the subsequent rebound epidemic was 0.62 and 0.88, respectively. The average resilience of the highway transportation system in Hunan Province during the two epidemic waves was 0.75. Comparing these values with the qualitative assessment of system resilience 0.72 in Section 3.2, it can be concluded that the qualitative assessment of system resilience and the quantitative analysis of the case data are consistent, effectively reflecting the resilience level of the highway transportation system during the epidemic.

**Table 5:** Comparison of system resilience during two epidemic waves.

	Index	Percentage decrease in system performance (%)	Indicator resilience	Overall resilience	Average system resilience
First wave of COVID-19 (January 23, 2020)	Freight volume	65.44	0.73	0.62	0.75
	Freight turnover	65.14	0.74		
	Passenger volume	84.79	0.47		
	Passenger turnover	68.43	0.53		
Rebound of COVID-19 (July 28, 2021)	Freight volume	5.09	0.97	0.88	
	Freight turnover	4.69	0.98		
	Passenger volume	38.17	0.82		
	Passenger turnover	48.70	0.76		

When the first wave of the COVID-19 pandemic outbreak, conventional emergency plans in the past have failed, panic caused by the huge unknown, and strict epidemic prevention and control measures on a large scale caused a sharp decline in population mobility, and the highway transportation system was on the brink of stagnation. After the outbreak of the first wave of the pandemic, the country and various regions continuously explored their experiences and began to implement more precise and differentiated measures for epidemic prevention and control, such as strengthening education about health and safety, tracking close contacts based on big data, targeted closure management, etc. Scientific and precise epidemic prevention has strengthened the system’s resilience. Therefore, the impact on the highway transportation system significantly decreased during the subsequent rebound of the epidemic. From this, we can find that to ensure the normal operation and sustainable development of the highway transportation system, prevention and rehearsals should be added to daily life against various emergencies. Of course, continuous learning and experience summarization are also essential to cope with more external disturbances in the future.

## 5. Conclusions

Based on the analysis of the formation dimensions and emergence process of safety resilience in highway transportation systems, we constructed a network hierarchical model of safety resilience for highway transportation systems, designed an evaluation index system of safety resilience for highway transportation systems, and further proposed an evaluation method of safety resilience based on fuzzy analytic hierarchy process. Finally, the effectiveness of the resilience evaluation method proposed was verified through examples of public health events. This provides a method and approach for accurately and efficiently evaluating the safety resilience of highway transportation systems and a decision-making basis for improving the system’s management capabilities of safety production and safety resilience level. The main conclusions are summarized as follows:

(1) The network hierarchical model of the safety resilience in the highway transportation system constructed helps to clearly identify the various levels involved in system resilience, evaluate and optimize system safety resilience hierarchically, and systematically carry out collaborative linkage and resilience management, providing reference support for emergency coordination of highway transportation in emergencies.
(2) The evaluation index system and evaluation method proposed for the safety resilience of highway transportation systems can better reflect the overall resilience of highway transportation systems during the epidemic, which is basically consistent with the results of quantitative data analysis. The safety resilience assessment method for highway transportation systems based on FAHP covers five levels of qualitative resilience evaluation, from strong, relatively strong, general, and relatively weak to weak. It has the advantages of flexibility and simplicity in solving unstructured decision-making problems in highway transportation, which helps to improve the safety production management and safety resilience level of highway transportation systems.
(3) From a micro perspective, the level of resilience shows a fluctuating state. The resilience assessment method for highway transportation systems based on FAHP can reflect the overall level of the system resilience from a macro perspective but cannot analyze the evolution of system resilience in detail, such as the speed and degree of system performance decline during the interference phase; the recovery speed and recovery capability of system performance during the recovery phase.

Overall, the paper analyzed the effectiveness of the resilience assessment method proposed, which can help assess the safety resilience of highway transportation systems accurately and quickly. But, there are some limitations to the study. First, this paper aims to propose a resilience assessment method that reflects the overall level of system resilience in emergencies at a macro level. By constructing a system resilience curve and analyzing examples, the effectiveness of the resilience evaluation method is verified. However, this method can only reflect the overall level of resilience and cannot provide a detailed analysis of system performance changes during the interference phase(such as the degree and speed of system performance decline, the degree of recovery, and recovery speed, etc.); Second, the COVID-19 pandemic, a global public health emergency, was chosen as the research scenario for resilience analysis. However, this research scenario is not universally applicable. In the future, the scope of the research scenario can be expanded to explore the changes in the safety resilience of highway transportation systems in general emergencies. At the same time, the research area can be expanded to compare the resilience levels of different cities and regions horizontally.

## Supporting information

S1 File (DOC)
