# Supplementary material for: Enhancing highway transportation safety resilience during emergencies: A network-based analysis and assessment
Source: PLoS One. 2024 Jul 19;19(7):e0307233. doi: 10.1371/journal.pone.0307233 (PMC11259266; doi:10.1371/journal.pone.0307233)
Supplement: S1 File — (DOC) [file pone.0307233.s001.doc]

**All raw data used in the paper**

**1.The importance of resilience indicators from experts**

**T**


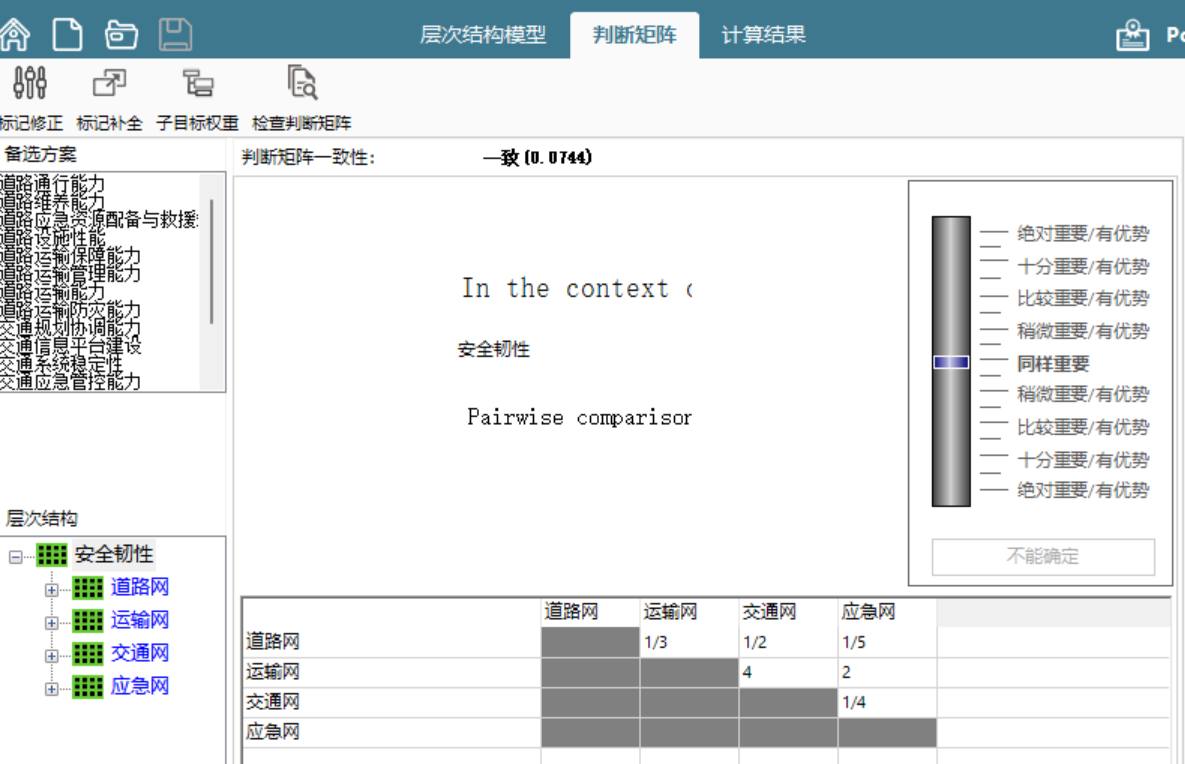


**T_1_**


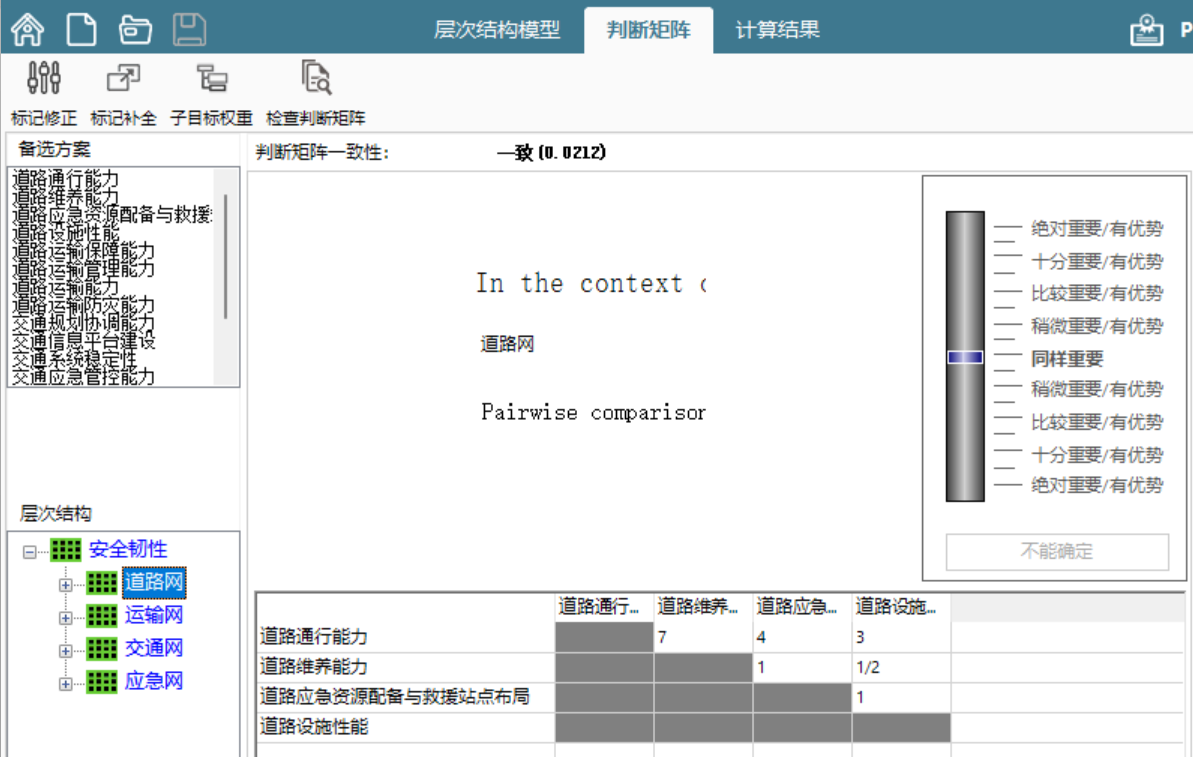


**T_2_**


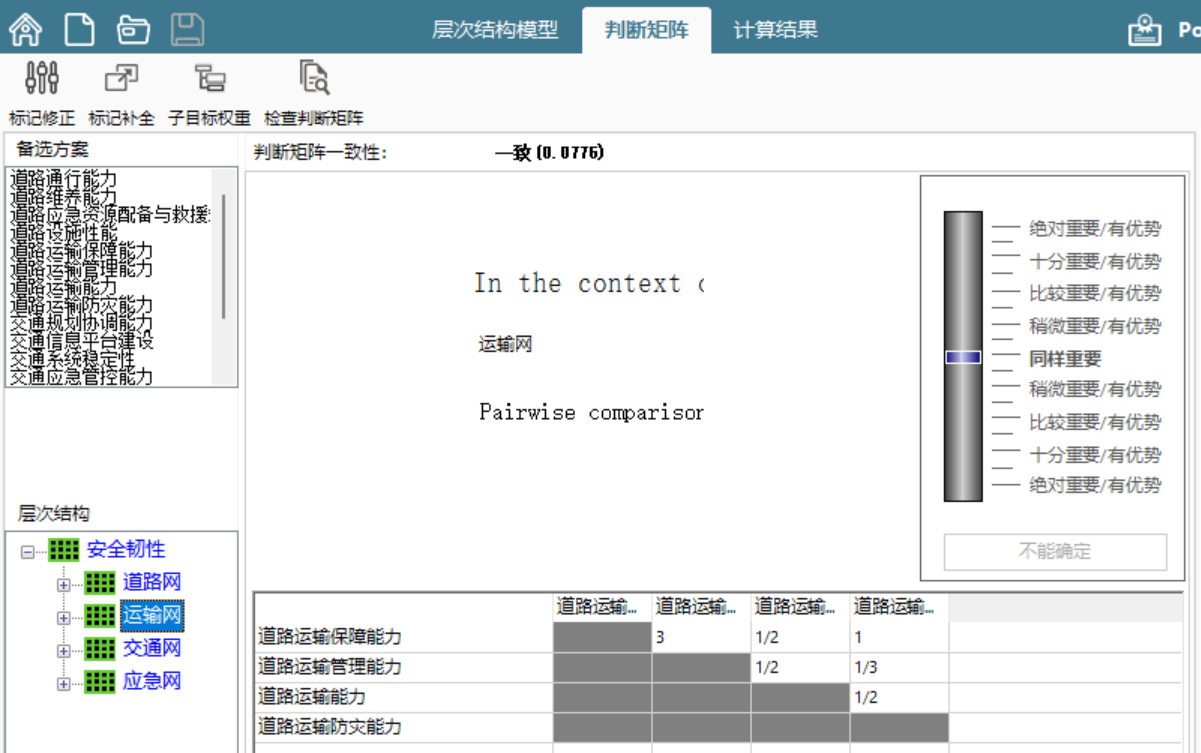


**T_3_**


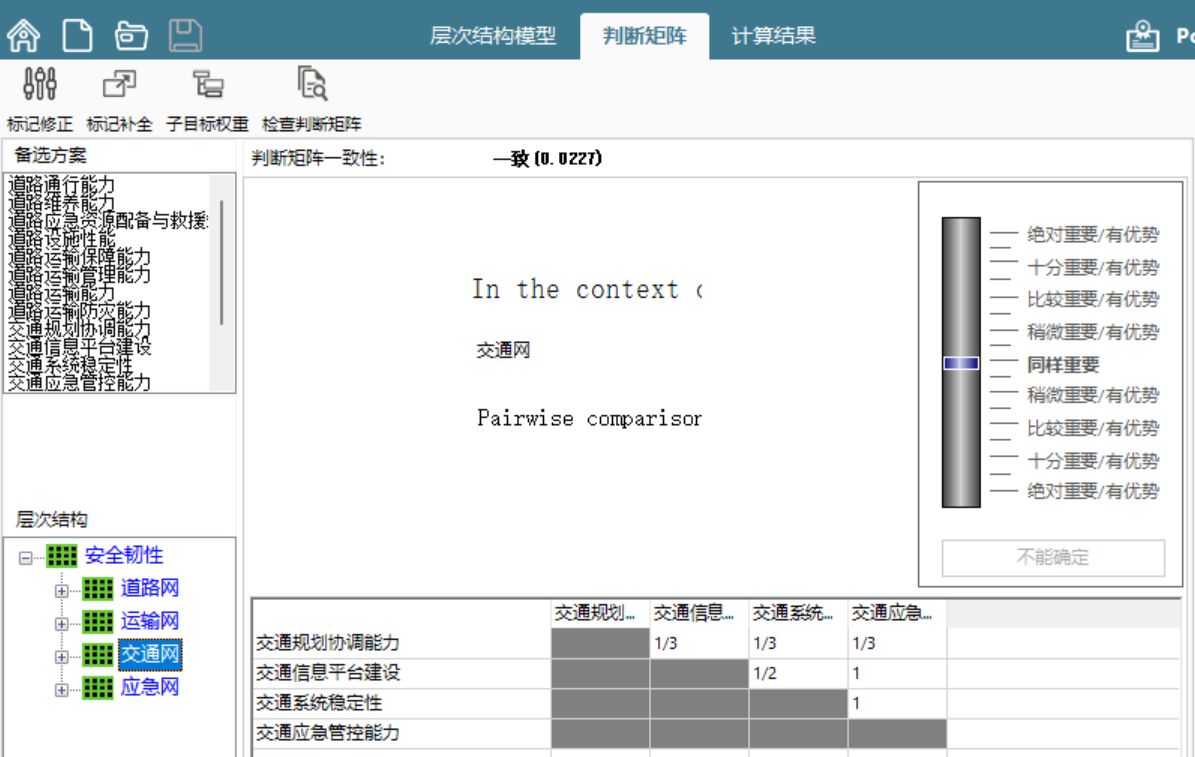


**T_4_**


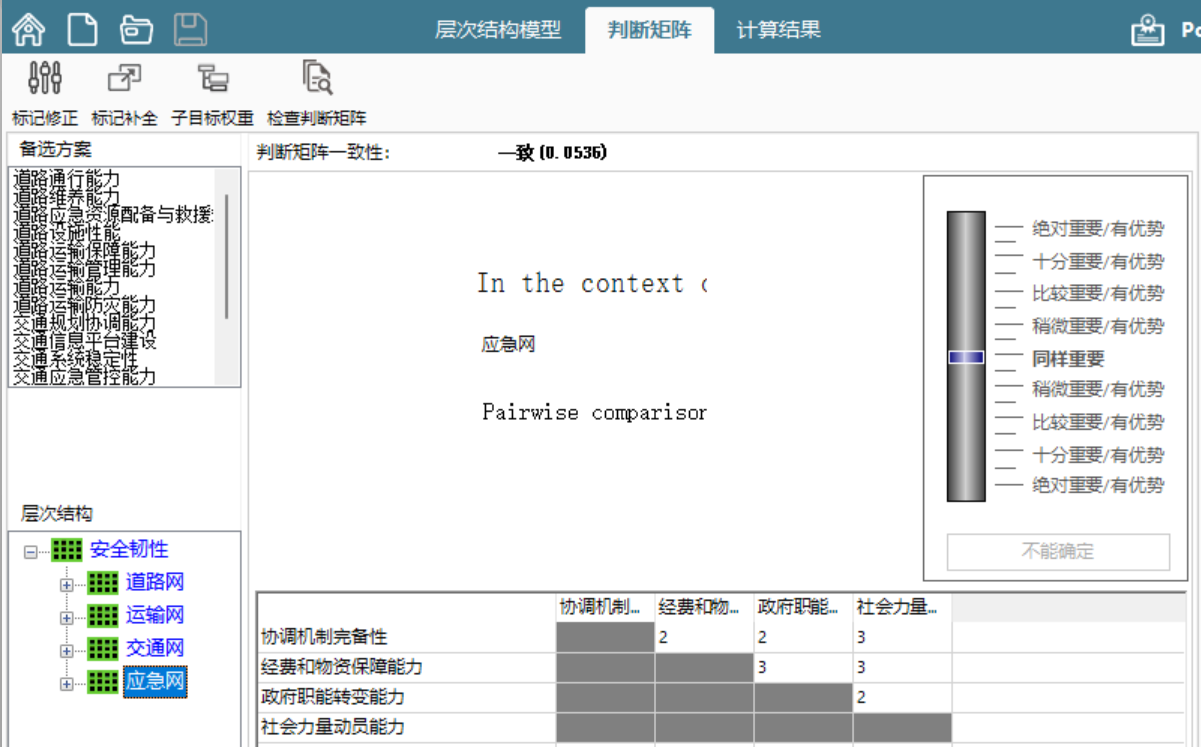


**2. Frequency statistics on the five-level resilience evaluation of indicators from experts**

| **Order number** | **Secondary indicators** |  | **Frequency** | | | | |
| --- | --- | --- | --- | --- | --- | --- | --- |
|  |  |  | strong | relatively strong | general | relatively weak | weak |
| 1 | Highway Traffic Capacity | t_11_ | 3 | 16 | 9 | 1 |  |
| 2 | Highway Maintenance Capability | t_12_ | 2 | 9 | 11 | 6 | 1 |
| 3 | Layout of Emergency Resources and Rescue Sites | t_13_ |  | 6 | 14 | 6 | 3 |
| 4 | Performance of Highway Facilities | t_14_ | 3 | 13 | 11 | 2 |  |
| 5 | Highway Transportation Security Capability | t_21_ | 8 | 11 | 10 |  |  |
| 6 | Highway transportation Management Capability | t_22_ | 10 | 19 |  |  |  |
| 7 | Highway transportation Capability | t_23_ | 4 | 18 | 6 | 1 |  |
| 8 | Highway Disaster Prevention Capability | t_24_ | 9 | 14 | 6 |  |  |
| 9 | Traffic Planning and Coordination Capability | t_31_ | 3 | 4 | 11 | 9 | 2 |
| 10 | Construction of Traffic Information Platform | t_32_ | 10 | 14 | 4 | 1 |  |
| 11 | Stability of Traffic System | t_33_ | 4 | 11 | 11 | 3 |  |
| 12 | Traffic Emergency Control Capability | t_34_ | 3 | 12 | 11 | 3 |  |
| 13 | Coordination Mechanism Completeness | t_41_ | 4 | 6 | 10 | 7 | 2 |
| 14 | Funding and Material Support Capability | t_42_ |  | 8 | 15 | 5 | 1 |
| 15 | Government Function Transformation Capability | t_43_ | 2 | 7 | 10 | 6 | 4 |
| 16 | Mobilization Capability of Social Forces | t_44_ | 8 | 9 | 10 | 2 |  |

1. Original monthly data of highway transportation in Hunan Province

| **Time** | **Freight volume (10000 ton)** | **Freight turnover (10000-ton kilometers)** | **Passenger volume (10000 person)** | **Passenger turnover (10000-person kilometers)** |
| --- | --- | --- | --- | --- |
| Jan-2019 | 14222.41 | 1157023.06 | 6514.57 | 307000.00 |
| Feb-2019 | 6955.47 | 565006.93 | 9670.40 | 561600.00 |
| Mar-2019 | 12473.69 | 1070409.87 | 6689.50 | 340900.00 |
| Apr-2019 | 13664.37 | 1115152.33 | 6625.06 | 317900.00 |
| May-2019 | 13354.96 | 1067748.84 | 6720.55 | 317300.00 |
| Jun-2019 | 13665.34 | 1009992.31 | 6379.87 | 315500.00 |
| Jul-2019 | 13456.19 | 1015882.47 | 7023.48 | 365700.00 |
| Aug-2019 | 13320.14 | 1062020.96 | 7316.87 | 397900.00 |
| Sept-2019 | 14810.08 | 1165448.50 | 7223.78 | 387100.00 |
| Oct-2019 | 14528.64 | 1165385.89 | 7806.93 | 440800.00 |
| Nov-2019 | 16496.04 | 1314254.03 | 6284.02 | 316300.00 |
| Dec-2019 | 18106.00 | 1454930.19 | 5906.97 | 266700.00 |
| Jan-2020 | 13553.96 | 1103800.00 | 7018.50 | 326200.00 |
| Feb-2020 | 4553.13 | 389200.00 | 1071.49 | 79000.00 |
| Mar-2020 | 11708.20 | 944800.00 | 1998.25 | 129100.00 |
| Apr-2020 | 14567.06 | 1165000.00 | 2648.77 | 133700.00 |
| May-2020 | 14529.09 | 1138700.00 | 3372.40 | 172000.00 |
| Jun-2020 | 15053.11 | 1094200.00 | 3135.76 | 159500.00 |
| Jul-2020 | 15232.55 | 1074500.00 | 3543.22 | 189600.00 |
| Aug-2020 | 16162.19 | 1161100.00 | 3723.92 | 201800.00 |
| Sept-2020 | 16359.48 | 1194300.00 | 4447.27 | 222800.00 |
| Oct-2020 | 15776.88 | 1253200.00 | 5179.17 | 266500.00 |
| Nov-2020 | 18709.35 | 1435400.00 | 4071.58 | 189800.00 |
| Dec-2020 | 20237.00 | 1551300.00 | 3933.67 | 178400.00 |
| Jan-2021 | 18088.00 | 1407003.00 | 4235.00 | 193294.00 |
| Feb-2021 | 7705.00 | 593169.00 | 3242.00 | 185855.00 |
| Mar-2021 | 16391.00 | 1201642.00 | 3206.00 | 185309.00 |
| Apr-2021 | 16228.00 | 1193463.00 | 3375.00 | 179593.00 |
| May-2021 | 17403.00 | 1262120.00 | 3608.00 | 197945.00 |
| Jun-2021 | 17473.00 | 1252667.00 | 3027.00 | 159972.00 |
| Jul-2021 | 17590.00 | 1265664.00 | 3356.00 | 193086.00 |
| Aug-2021 | 16598.00 | 1201073.00 | 2059.00 | 94223.00 |
| Sept-2021 | 17686.00 | 1276652.00 | 2913.00 | 150269.00 |
| Oct-2021 | 16861.00 | 1215280.00 | 3165.00 | 172400.00 |
| Nov-2021 | 17420.00 | 1255311.00 | 2431.00 | 119901.00 |
| Dec-2021 | 18980.00 | 1487556.00 | 2414.00 | 121953.00 |
